# COVID-19 Related Shifts in Social Interaction, Connection, and Cohesion Impact Psychosocial Health: Longitudinal Qualitative Findings from COVID-19 Treatment Trial Engaged Participants

**DOI:** 10.3390/ijerph191610264

**Published:** 2022-08-18

**Authors:** Amaya Perez-Brumer, Rebecca Balasa, Aarti Doshi, Jessica Brogdon, Thuy Doan, Catherine E. Oldenburg

**Affiliations:** 1 Dalla School of Public Health, University of Toronto, Toronto, ON M5T 3M7, Canada; 2 Children’s Hospital of Eastern Ontario Research Institute, Ottawa, ON K1H 5B2, Canada; 3 Francis I. Proctor Foundation, University of California, San Francisco, CA 94158, USA; 4 Department of Ophthalmology, University of California, San Francisco, CA 94158, USA; 5 Department of Epidemiology & Biostatistics, University of California, San Francisco, CA 94158, USA; 6 Institute for Global Health Sciences, University of California, San Francisco, CA 94158, USA

**Keywords:** COVID-19, social connection, social support, psychosocial health

## Abstract

While effective for slowing the transmission of SARS-CoV-2, public health measures, such as physical distancing and stay-at-home orders, have significantly shifted the way people interact and maintain social connections. To better understand how people sought social and psychological support amid the pandemic, we conducted a longitudinal qualitative evaluation of participants enrolled in a COVID-19 treatment trial (*N* = 30). All participants from the parent trial who consented to being contacted for future research studies were recruited electronically via email, and first-round virtual interviews were conducted between December 2020 and March 2021. Participants who participated in first-round interviews were contacted again, and follow-up interviews were conducted in January–February 2022. The results reported significant shifts in how participants connected to social support, including changes from physical to virtual modalities, and using different social networks for distinct purposes (i.e., Reddit/Facebook for information, WhatsApp for community connection). While having COVID-19, profound loneliness during isolation was described; yet, to mitigate effects, virtual support (i.e., emotional, knowledge-seeking) as well as in-person material support (e.g., groceries, snow-shoveling), were key. Public health efforts are needed to develop interventions that will improve the narratives about mental health challenges related to COVID-19 isolation, and to provide opportunities to share challenges in a supportive manner among social networks. Supporting social cohesion, despite the everchanging nature of COVID-19, will necessitate innovative multimodal strategies that learn from lived experiences across various stages of the pandemic.

## 1. Introduction

The COVID-19 pandemic has had a devastating and unprecedented impact on psychological wellbeing globally [1,2]. Amid the pandemic, the United States (US) has observed an estimated 29.2% to 35.0% increase in the prevalence of major depressive disorder, and an estimated 25.6% to 28.8% increase in the prevalence of anxiety disorders [1]. The pandemic’s burden on mental health is associated with multiple intersecting factors, including economic decline resulting in increased job insecurity [3,4]; housing precarity [5,6]; childcare disruptions and school closures [7,8,9]; increased risk of interpersonal violence [10,11]; and general disruption to daily routines [12]. Furthermore, the burden of COVID-19 disease, and its psychosocial sequelae, has disproportionately impacted certain populations, such as children and youth [12,13], cisgender women [14,15,16], minoritized communities [17,18], people who use drugs [19], older adults [20,21], people with disabilities [22,23], and LGBTQIA+ individuals [24,25,26,27].

While physical distancing and isolation requirements (i.e., quarantine) have been key public health measures for slowing the transmission of SARS-CoV-2 (COVD-19) [26,28,29,30,31], these measures have had unintended negative consequences for psychosocial health. Quarantine and physical distancing policies amid COVID-19 have exacerbated social isolation [32,33]. These implemented policies have reportedly increased loneliness, anxiety, and depression [34,35]; they have increased feelings of helplessness surrounding COVID-19 transmission and health sequelae [36]; and they have increased substance use to cope with COVID-related stress amid the pandemic [37]. While there is much evidence linking social isolation and loneliness to worsening mental health outcomes [38], scant literature has explored how people with COVID-19 must shift their modalities of social connection to follow public health guidelines, and the resulting psychosocial impacts.

Moreover, the impact on social relations, of maintaining physical distancing and isolation requirements, and the resulting negative mental health outcomes, have not equally impacted groups and individuals in society [2,39,40,41]. Limitation of access to appropriate technology to maintain social interactions (e.g., no internet service, no electronic device(s), no support person/mediator) has led to greater social exclusion among those experiencing such technological disparities [39]. Yet, research exploring how people have experienced these changes in social relations—specifically new forms of interactions, connections, and impacts on social cohesions—has been limited. More nuanced assessments are needed, of the ways in which COVID-19 prevention and control measures may have impacted how people create new, and maintain existing, social connections and support psychosocial wellbeing.

Lessons from the H1N1 influenza pandemic of 2009 demonstrated that social support is important for coping with, and recovery from, loneliness and isolation [42,43]. In the COVID-19 context, emerging literature has evidenced that the increase in social connections, amid waves of lockdowns and enforced distancing, has resulted in notable shifts in modalities for social interaction rooted in increased technology usage [44]. Video conferencing software (e.g., Zoom, Google classrooms), private text-based platforms (e.g., iMessage, WhatsApp), and social media outlets (e.g., Instagram, Twitter) have allowed individuals to connect regardless of geographical constraints [45,46]. These shifts have included the transition of cultural settings into virtual spaces such as virtual classrooms [47], virtual places of worship [48], and virtual communication platforms for social TV viewers [49].

Given the dynamic and ongoing impacts of the pandemic, there is a need to assess the longitudinal shifts in social connections, and to examine the impact of these shifts on psychosocial wellbeing. To explore these shifting social connections over time, and their impacts on material and psychosocial support, we conducted a longitudinal qualitative evaluation of participants enrolled in a COVID-19 treatment trial.

## 2. Materials and Methods

### 2.1. Research Design

The data presented here were drawn from a longitudinal qualitative evaluation of a nation-wide randomized controlled trial (RCT) study (i.e., *ACTION*), examining the use of Azithromycin for mild-to-moderate symptoms of COVID-19 [50]. This qualitative study involved interviews conducted across a one-year interval (December 2020–March 2021 and January–February 2022), to explore the experiences and perspectives of social connectedness among participants who were previously diagnosed with COVID-19 and engaged in an RCT amid the pandemic.

### 2.2. Data Sampling and Recruitment

At the first timepoint, between December 2020 and March 2021, participants from the ACTION study who consented to being contacted for future research studies, were recruited electronically via email. Enrollment was limited to ACTION study participants, aged 18 and older, who met the following criteria: (1) they had received a positive COVID-19 test result; (2) they resided in the US; (3) they had obtained capacity to consent. Paralleling the larger study, no economic compensation was provided for participation in the qualitative evaluation. All participants signed a written informed consent, and ethics approval was obtained for all study procedures from the University of California, San Francisco (IRB protocol #20-32453) and the University of Toronto (REB protocol #23729).

### 2.3. Data Collection and Analysis

All interviews were between 20–50 min in total, and were audio-recorded using Zoom software. Nineteen participants were interviewed at timepoint 1 (T1) between December 2020 and March 2021. After being contacted again for a second interview, 11 participants were interviewed at timepoint 2 (T2) between January and February 2022. A semi-structured interview guide was used to question the participants about their experiences in a remote clinical trial during the COVID-19 pandemic, about ongoing forms of social support, and about their required access to healthcare. During the interview, participants were randomly allotted a participant identification number. All de-identified audio-recordings of the participant interviews were transcribed verbatim by a professional transcriptionist.

Data from the transcripts were analyzed using immersion crystallization, an inductive and deductive method of identifying parent themes and child themes within the coding process [51]. This analytic approach entailed close reading of transcripts, coding subsets of transcripts through an iterative process, organizing the codes, thematic development and discussion, and hierarchical organization of the themes. This iterative approach emphasized examining data and reflecting on the experience of being immersed in the data analysis at two timepoints, to improve analytic comparison across stages. Analytic memos were used through the analysis and writing process, as analytical devices to engage in a value-adding analysis [52]. We double-coded 25% of the transcripts, to ensure a consistent approach, and any coding differences encountered were discussed and reconciled at regular team meetings. Dedoose Version 9.0.17, a web application for managing, analyzing, and presenting qualitative and mixed method research data, assisted the analyses (2021); Los Angeles, CA, USA: SocioCultural Research Consultants, LLC.

## 3. Results

At T1, we conducted 19 interviews, and at T2 we conducted 11 interviews with a sub-set of participants from T1. The mean age for participants across interviews was 47 years old, and the majority (63%) were women and self-identified as white (75%). Most participants resided in California (58%). See Table 1 for additional demographics. The main results are presented across timepoints, and are organized in three key themes: (1) shifting modalities for social connection amid COVID-19; (2) limits and adjustments to shifting forms of social interaction; (3) ‘New normal’: reflections of the ongoing impact of COVID-19 on social cohesion.

### 3.1. Shifting Modalities for Social Connection Amid COVID-19

Between December 2020 and March 2021 (T1), all participants described changes to how they connected with social networks, namely noting a shift from physical to virtual platforms. Most participants described the composition of the core members of their social support networks as being comprised of family, friends, neighbors, and/or colleagues. In consideration of public health measures to slow the transmission of COVID-19, such as physical distancing, numerous participants detailed being “busy texting everybody, WhatsApp-ing everybody” (T1, 56 y/o, W, CA) and “keep[ing] up with [others] over Zoom or on the phone” (T1, 35 y/o, M, CA). Another participant noted the advent of frequent and routine check-in messages, such as to “[reach out] if [they] need[ed] anything” and/or “writ[ing] to [friends/family] every day” to check in (T1, 50 y/o, W, CA). Other participants explored social interactions and connections through “outdoor play dates or hangouts” (T1, 37 y/o, M, CA), such as “hang[ing] out at parks or something every other week” with social networks that they felt comfortable meeting in person (T1, 35 y/o, M, CA).

Several participants noted the intense use of virtual platforms early in the pandemic, to provide social and emotional support to members of their existing familial and friend networks. While the increase of connecting via virtual forums, to maintain social connections, was frequently described, participants noted that, as the pandemic progressed, so did their use of various platforms to accommodate the needs of different networks. For example, participants described utilizing group-based chat modalities for close friends and family (i.e., WhatsApp), larger thread response platforms for more extended networks (i.e., Facebook, NextDoor), and formal corporate platforms (i.e., Microsoft Teams, Slack) with colleagues.

Notably, all participants in our study had COVID-19, and recounted the multiple ways that they received various forms of support via their social networks. One participant detailed how her bible study group had both a virtual group chat and phone tree system to ensure their community had the resources they needed. When she and her husband tested positive for COVID-19, their bible study group “took [the] initiative to […] set up a schedule [for delivering groceries]” during their isolation period (T1, 74 y/o, W, CA). Cautionary informational support from social networks regarding COVID-19 sequelae were also detailed by participants: “My friends warned me […], ‘Look, we’ve read, and we’ve heard, day seven is the worst—it gets worse. You think you’re getting better, then it gets worse.’ Sure enough, that’s when the breathing issues came in” (T1, 50 y/o, W, CA). Furthermore, a few participants with access to friends or family who were healthcare providers, also described their “ab[ility] to [just] call” with any question or concerns related to their symptoms (T1, 49 y/o, W, GA). Importantly, shifts in social connection were also noted beyond the virtual domain, with many participants recounting being more aware of their neighbors and neighborhood. One participant described the efforts of her neighbors, who knew she was recovering from COVID-19, and who therefore shoveled her driveway: “We had a big snowstorm while I was in isolation and somebody came and shoveled me out in case I needed to get out, which thankfully they did ‘cause the next day I went to the [emergency room]” (T1, 49 y/o, W, NY).

At T2, following a year of new COVID-19 variants and associated lockdowns, between January and February 2022, some participants highlighted waning enthusiasm and demand for, and upkeep of, virtual forms of social connections, compared to earlier in the pandemic. Some participants described having a newfound connection with the people that they were residing with, yet having difficulty maintaining connections with their larger social networks. For example:

“[Our family is] much closer. Because we all had to be home together, and not run crazy and hang out together, and that was a really positive dynamic for our family in that we realized that our family is more important and that relationships are more important. But I’m finding with friends and acquaintances that I personally need to make more time to meet them for lunch, as opposed to like a Zoom call. Like my book club are like, ‘Let’s just meet for Zoom, it’s easier,’ and I’m like yeah, but you miss that connection of getting together and having a glass of wine and talking about it“(T2, 49 y/o, W, GA).

Participants also described ongoing challenges regarding when to transition back to in-person socialization. Participants provided justification for in-person versus virtual gatherings during this period, explaining how “we are all social beings. And we need to interact with other people—not only [via] Zoom but also in-person” (T2, 74 y/o, W, CA). Leveraging what they learned about their networks, participants described being selective in who they saw in-person: “I know pretty much everybody that I hang out with is vaccinated, and if they weren’t vaccinated, I would probably not hang out with them” (T2, 56 y/o, W, CA). However, participants also anticipated the continuation of some pandemic-specific social norms: “a lot of [it] is going to be a shift towards less in-person socialization and increased uptake of video socialization or text messaging, you know, social media, that kind of thing” (T2, 32 y/o, M, CA). Further, one participant, who lived in Hong Kong amid SARS, shared her parallel experience of what a new ‘normal’ for social connection might entail:

“In my experience with SARS in Hong Kong, it was short lasting. This time [with COVID-19], [it’s] very long. And [we’ve gotten] pretty impatient about—oh we want to get back to normal life […] I think now we have the habit of using Zoom. And people work at home more. So, some companies, some agencies don’t rent the office anymore. They just have everybody work from home. So, these things that we’ve kind of had to do to adapt to COVID […] I call [them] the new normal” (T2, 74 y/o, W, CA).

### 3.2. Limits and Adjustments to Shifting Forms of Social Interaction

All participants reflected on how social connection changed amid COVID-19, yet perspectives differed in regard to how these shifts impacted them, especially while they had COVID-19. This section details the challenges described, in regard to the shifting and new ways of promoting social interaction and psychosocial wellbeing. Behavioral responses from social networks, associated with the initial shock of disclosing a COVID-19 positive status, were detailed by many participants: “First everybody flood[s] you with phone calls. ‘Are you okay?’ This, that, the other. It’s almost to the point where it’s like, ‘Okay. Just text me with the thumbs up that you’re doing okay’ because, of course, you’re [still] isolated in your house” (T1, 51 y/o, W, CA). Some participants described social supports as “very supportive but also very curious”, subsequently probing on “‘How did [the COVID-19 infection] happen? What did you feel like? What was different the first time versus the second time, you know, that kind of thing” (T1, 54 y/o, W, MI).

Despite expressing gratitude for “ha[ving] people calling and checking up on [them]”, one participant described distanced social supports as “not the same when you [already] feel alone and isolated... feeling imprisoned when you’re not in prison” (T1, 41 y/o, W, CA). Divergences in beliefs, and abilities to physically distance, were also reported by some participants as creating rifts in social networks. One participant mentioned being assigned blame for their COVID-19 positive status, explaining how members of their social networks often stigmatized them “because it’s easy for people, if you’re infected, to assume you misbehaved” (T1, 71 y/o, M, CA). Other experiences of stigma were noted by a participant whose social network denied their experiences of testing positive for COVID-19 due to the politicization of the COVID-19 pandemic: “There’s a lot still from the political end of it […] I had somebody who, after I’ve gotten over [COVID-19], just recently say, you know, ‘it was a man-made’ or ‘it was a hoax’” (T1, 49 y/o, W, NY). Several other participants echoed these sentiments of feeling anxiety and shame to share their COVID-19 status with close networks, for fear of stigma. As a result, many sought anonymous support via larger forms of online networks (i.e., Reddit, Facebook).

When describing what it was like having COVID-19 early in the pandemic, participants described that they felt that their discomfort and, for some, fear, was exacerbated due to the limited and contradictory information available. To connect with other people who also had a COVID-positive diagnosis, and share resources, many participants joined “Facebook COVID support groups”, which they felt “was a good thing and a bad thing, because it was very supportive and informative, but it [was] also scary because [of the] horror stories” (T1, 50 y/o, W, CA). Similarly, many participants recounted that, while isolating due to having COVID-19, they went searching down online rabbit holes. For many, this process exacerbated anxiety and extreme paranoia. One participant noted, “My husband says I’m not allowed to Google it anymore because of course I think I have every symptom. Uh, you know, you worry about like, ‘Could it cause dementia? Could it have long-term heart problems or lung problems?’” (T1, 49 y/o, W, GA).

Most participants noted that their closest social interactions were with people they lived with. Despite many participants describing the positive emotional social support within their own household, some participants spoke about the perceived challenges as well. In reference to their Indian future daughter-in-law’s family structure, one participant recounted the observed difficulties of physical distancing within multi-generational households that were upheld by collectivist cultures, whereby “all 12 people in the household got the virus” and, thankfully, “all recovered” (T2, 71 y/o, M, CA). Primal fears associated with COVID-19 transmissibility within the household were also described in reference to immunocompromised populations, including the need to address the compounded psychosocial impacts experienced by these populations while receiving treatment: “Some [cancer patients] are being tested positive, some of them are living with family members that are being tested positive, and then they’re also going through chemotherapy. […] So, it’s just a lot of […] emotional care giving that we’re giving as well” (T1, 51 y/o, W, CA).

### 3.3. New Normal’: Reflections of the Ongoing Impacts of COVID-19 on Social Cohesion

Specific to T2, January–February 2022, participants spoke of various psychosocial impacts associated with the barriers and facilitators of transitioning back to in-person forms of social interaction, described by some as the “new normal”. Central to these descriptions were frustrations about people of certain political affiliations, as perceived by participants. One participant described needing to “limit [their] time” with certain family members, due to the exhaustion associated with rationalizing opposing views: “Like [my partner]’s parents are in their 60 s […] but they also live in a red county, and they’re also uneducated. So, which of those factors is the reason that they won’t put a fucking mask on when they’re in public, I have no idea” (T2, 47 y/o, W, MO). Another participant recounted one tweet from a friend who stated: “I see how you act in a pandemic, and you and I will continue to be friends or not continue to be friends based on this. I will know, as much as I need to know about you, based on the way you acted during this whole thing” (T2, 56 y/o, W, CA).

Participants also reported greater psychosocial concerns relating to the ongoing public health measures that limited in-person gatherings, explaining that “it’s definitely meant that life has been a little bit more lonely. Just limiting the number of people that you are seeing” (T2, 32 y/o, M, CA). Looking forward to the future, participants “would be content with life if [they could] see [other people’s] faces, if [they] didn’t have to worry about going grocery shopping, if [they] could watch a basketball game with [their] family and friends and not have to worry about who has or hasn’t had a shot” (T2, 39 y/o, W, NY).

Participants narrated that the ways people now interact and connect have fundamentally shifted. One participant illustratively stated the importance of psychosocial support while COVID-positive: “After going through all [of] this, one of the things that I’ve learned is how important it is to have social support... from your family, from your friends who love you. And I think this is very, very important. Without them, I think it’s very hard to go through [the] experience [of having COVID-19]” (T1, 74 y/o, W, CA).

Indeed, many participants noted positives, such as an increase in telehealth and ongoing video calls with elder relatives. Yet, most also voiced the impact of experienced and perceived inequities rooted in differential access to the supports and resources required to physically distance and engage in sustained virtual social networks. For example, one participant, a teacher, noted how the transition to virtual classrooms led to increased barriers, for the students, to maintaining psychosocial wellbeing while isolating:

“It’s hard to contact [the kids who don’t have internet at home] so they can stay up on their studies. I know there’s children who don’t have heat or running water at home. And I know that food is an issue for them because they rely on the fact that our entire school district qualifies for free lunch and breakfast every day, and when your home for five to ten days straight, that’s ten meals you’re not getting” (T1, 39 y/o, W, NY).

Further, when reflecting on the broad psychosocial impacts of the shifts in social interaction, connection, and cohesion, many participants described having a close friend or family member that was experiencing and navigating extreme mental illness. For example, “I’ve had friends who have kids that are in the pre-teens who have ended up with a mental illness, suicide, attempted suicide. And it’s because they’ve been isolated and kids are—you know, humans are—we’re creatures—we are creatures that need each other, and now you’re isolating a child who needs that interaction” (T2, 51 y/o, W, CA). Furthermore, across timepoints, participants underscored that the ways people now interact and connect have fundamentally shifted. One participant illustratively stated the importance of psychosocial support while COVID-positive:

“After going through all [of] this, one of the things that I’ve learned is how important it is to have social support... from your family, from your friends who love you. And I think this is very, very important. Without them, I think it’s very hard to go through [the] experience [having COVID-19]” (T1, 74 y/o, W, CA).

## 4. Discussion

This study contributes new knowledge on how people who have had COVID-19 engage with and create new social networks, as well as the psychosocial impacts for this unique group. In this longitudinal study, participants describe the entanglement between the need to engage in social connection while balancing between using virtual platforms and navigating a return to in-person activities. The needs of social interaction and connection varied across the sample, and changed based on the degree of fulfillment of emotional and material support needs (i.e., limited information, isolation, recovering from COVID-19). Not all forms of social interaction and connection yielded social cohesion, but when they did, the contributory relationship between social cohesion and improving the impacts of isolation and psychosocial wellbeing were detailed among participants.

Efforts to facilitate and maintain social connections amid COVID-19 were reported as dynamic across different phases of the pandemic. The findings underscore early enthusiasm for using virtual forms of social media, and video-chatting technology, to ‘check in’ during the initial exogenous shock caused by mass lockdown measures. Although participants highlighted that maintaining social connection amid the COVID-19 pandemic was central to mitigating the detrimental impacts of isolation [53], abrupt shifts to virtual forms of social interaction were insufficient to foster social cohesion, when compared to pre-COVID physical interactions. While pre-existing strong social and familial bonds could be sustained and, for some, were fortified via online group chats, increased anxiety was described in regard to not being able to physically see and support others through the early stages of COVID-19. At timepoint 2, participants described the burden of, and waning enthusiasm for, keeping up the frequency of virtual social interaction across multiple arenas of daily life (i.e., family, friends, work). These findings suggest an added complexity, as the existing literature on social connection during the COVID-19 pandemic reports the detrimental effects of increased isolation due to physical distancing measures [38,54,55,56,57], and the potential mitigating benefits of virtual platforms as spaces for social connection [58,59]. Furthermore, this study provides insight into future research needed to better understand the changing dynamics of social connection across and beyond the stages of the COVID-19 pandemic.

It is important to underscore that all participants described varying forms of social connection as being key to seeking emotional and material support while they had, and recovered from, COVID-19. During this time, many described acute periods of anxiety at the nexus of isolation, virtual ‘rabbit holes,’ and heightened exposure to information about mortality and negative health outcomes associated with COVID-19. Supporting literature evidences virtual-information-seeking as a coping and maladaptive strategy during the early stages of COVID-19 [60,61]; our participants underscored that the continual drive to seek information about COVID-19 often fueled anxiety. Participants described a shift towards seeking medical and public health information via social networks and social media, due to the lack of information (especially prior to the roll-out of vaccines). This aligned with existing literature concerning the spread of misinformation during the COVID-19 pandemic, that demonstrated the complex associations between sourcing information from informal sources—such as social media and personal contacts—in fueling conspiracy theories and misinformation beliefs, subsequently heightening experiences of anxiety and depression [62,63]. Subsequent research is needed to better understand the relationship between demographics characteristics (e.g., age, race/ethnicity, geographic location) and primary sources of health-information-gathering (composition of social network [e.g., friends, family] and virtual platforms [Reddit, Facebook, etc.]) to inform future health literacy efforts based on COVID-19-related shifts.

Although many of those who participated between December 2020 and March 2021 expressed their gratitude for receiving material support while COVID-19 positive, the interviews conducted between January and February 2022 revealed variations in household composition, that influenced differential psychosocial needs among historically marginalized groups. This observation built on the existing literature highlighting the disproportionate psychosocial impacts experienced by marginalized populations due to COVID-19, and the measures implemented to mitigate its transmission [2,17,31]. Requirements to isolate from social support networks that provided survival needs (i.e., food, water, heating) were described as impeding viability and psychosocial development, particularly among school-aged children. Consistent with our findings, recent evidence has shown that individuals with lower socioeconomic status are more likely to possess fewer material resources with which to facilitate staying home during the COVID-19 pandemic; greater challenges have also been identified with physical distancing, if residing in densely populated households, increasing the risk of exposure to, and potential transmission of, COVID-19. Difficulties in maintaining physical distancing were also described in reference to multi-generational households with cultural norms rooted in collectivism (i.e., prioritizing the needs of the social group over the individual). Oftentimes, bearing familial obligations to provide psychosocial and financial support, family caregivers were shown to have experienced significant baseline symptoms of anxiety and heightened post-traumatic stressors, also evidencing an increased likelihood of socially retracting from networks, and subsequent experience of avoidance distress amid the COVID-19 pandemic [2,64].

Across both timepoints, all participants spoke extensively of multidimensional anticipatory stressors associated with navigating a ‘new normal’. While some participants expressed an immense desire to return to a pre-pandemic state of normalcy, others described feelings of existential anxiety in adapting to a ‘new normal’, whereby COVID-19 remained an ongoing concern. Stemming from the politicization of the COVID-19 pandemic [65,66], strong sentiments regarding shifts in perceived belongingness within, and tolerance towards, pre-existing social networks were detailed by participants whose beliefs aligned with continued adherence to public health measures. Other participants, however, detailed feelings of exhaustion from challenging behavioral responses that contradicted COVID-19 safety precautions among social networks, resulting in ‘limiting their time’ with respective members to preserve their psychosocial wellbeing. The longitudinal design of the interviews allowed us to speak to participants during two different presidential terms. While political orientation changed, description of politics was described as being already entrenched within networks, and linked to perceptions of failure to comply with health-protective behavior recommendations, and increases in contracting COVID-19. Even post vaccine roll-out, the decision voiced by many participants was to separate or to end these relations, which was described as leading to sadness, blame, and anxiety over broken social ties.

The findings presented here should be considered alongside their several methodological limitations. As the study sample was purposefully small, to allow for in-depth analysis and to enhance the validity of the inquiry [67], these qualitative findings are not generalizable, and thus may not be transferable to other settings. Our findings should be regarded as a first step towards a better understanding of the complex changes in how social connections have shifted, and continue to shift, amid COVID-19. Future studies should consider purposive sampling, not from within a nationwide trial, to better ascertain the impacts of race/ethnicity, precarity, and pre-existing mental health conditions. As noted, participants in this study were recruited from a larger, parent COVID-19 treatment study, and thus had the resources to participate in a remote trial, including stable housing, and access to phone and internet. Scholars seeking to further this line of research inquiry should undertake qualitative methods involving people with limited-to-no access to technology, and/or limited technological literacy, in insecure housing, to better assess shifts in social interactions, connections, and cohesions.

## 5. Conclusions

The findings of this study contribute an imperative understanding of the dynamic shifts in social connection, and the resulting psychosocial impacts, among people with a COVID-19 positive diagnosis, amid the pandemic. Given the importance of social cohesion in deterring isolation and supporting psychosocial wellbeing, public health efforts are needed to develop solution-focused language interventions that will improve how we speak about mental health challenges related to COVID-19 among social networks, so as to provide the opportunity to share worries and challenges in a supportive manner. While social interactions during the initial stages of the COVID-19 pandemic were substantive, in providing multidimensional support for many participants during isolation periods, the politicization of COVID-19 crucially limited social networks as collaborative spaces for support. Supporting social cohesion, despite the everchanging nature of COVID-19, will necessitate innovative multimodal strategies that learn from lived experiences across various stages of the pandemic.

## Figures and Tables

**Table 1:** Participant demographic characteristics.

Characteristic	N (%)
Race/Ethnicity ^l^	
African American	1 (5.3%)
Afro-Caribbean	1 (5.3%)
Asian American	2 (10.5%)
Black	1 (5.3%)
East Asian	2 (10.5%)
Hispanic	3 (15.8%)
Indian American	2 (10.5%)
South Asian	2 (10.5%)
Southeast Asian	2 (10.5%)
White	15 (75.0%)
Co-morbidities ^1^	
None	14 (73.7%)
Asthma	2 (10.5%)
Chronic kidney di sease	1 (5.3%)
Diabetes	1 (5.3%)
History of cancer	1 (5.3%)
Hypertension	2 (10.5%)
State	
California	11 (57.9%)
Georgia	1 (5.3%)
Michigan	1 (5.3%)
Missouri	1 (5.3%)
New Mexico	1 (5.3%)
New York	3 (15.8%)
Texas	1 (5.3%)

^1^ Sum is >100% because some participants selected more than one option.

## Data Availability

If interested in accessing original transcripts, please email request to corresponding author, Amaya Perez-Brumer (a.perezbrumer@utoronto.ca).
